# The Assessment of the Long-Term Impact of Radiotherapy on Biophysical Skin Properties in Patients after Head and Neck Cancer

**DOI:** 10.3390/medicina60050739

**Published:** 2024-04-29

**Authors:** Jakub Pazdrowski, Adriana Polańska, Joanna Kaźmierska, Michał J. Kowalczyk, Mateusz Szewczyk, Patryk Niewinski, Wojciech Golusiński, Aleksandra Dańczak-Pazdrowska

**Affiliations:** 1 Department of Head and Neck Surgery, Poznan University of Medical Sciences, 61-866 Poznan, Poland; mateusz.szewczyk@wco.pl (M.S.); niewinski.md@gmail.com (P.N.); wgolus@ump.edu.pl (W.G.); 2 Department of Head and Neck Surgery, Greater Poland Cancer Centre, 61-866 Poznan, Poland; 3 Department of Dermatology and Venereology, Poznan University of Medical Sciences, 60-355 Poznan, Poland; apolanska@ump.edu.pl (A.P.);; 4 Radiotherapy Department II, Greater Poland Cancer Centre, 61-866 Poznan, Poland; joanna.kazmierska@wco.pl; 5 Department of Electroradiology, Poznan University of Medical Sciences, 61-866 Poznan, Poland; 6 Department of Dermatology, Poznan University of Medical Sciences, 60-355 Poznan, Poland; aleksandra.danczak-pazdrowska@ump.edu.pl

**Keywords:** radiotherapy, radiation induced skin injury, radiodermatitis, skin barrier, high-frequency ultrasonography

## Abstract

*Background and Objectives:* Chronic radiotherapy-induced skin injury (cRISI) is an irreversible and progressive condition that can significantly impact a patient’s quality of life. Despite the limited literature available on the assessment of the epidermal barrier in cRISI, there is a consensus that appropriate skincare, including the use of emollients, is the primary therapeutic approach for this group of patients. The aim of this study was to evaluate the biophysical properties of the skin during the late period (at least 90 days) following radiation therapy (RT) for head and neck cancer. *Materials and Methods*: This was a single-center prospective non-randomized study. It involved the analysis of 16 adult patients with head and neck cancer who underwent RT at the Greater Poland Cancer Center, along with 15 healthy volunteers. The study and control groups were matched for gender and age (*p* = 0.51). Clinical assessment, based on the LENT-SOMA scale, was conducted for all patients. Evaluation of the skin’s biophysical properties included: an analysis of transepidermal water loss (TEWL), stratum corneum hydration (SCH), and skin visualization using high-frequency ultrasonography (HF-USG). *Results:* A significantly higher TEWL was observed in the irradiated area compared to the control area in the study group (*p* = 0.004). However, there was no statistically significant difference in SCH (*p* = 0.073). Additionally, no significant difference was observed in the values of TEWL and SCH in the irradiated area between the group of patients with and without clinically obvious RISI (*p* = 0.192 and *p* = 0.415, respectively). The skin thickness of the irradiated area, assessed by HF-USG, did not differ significantly from the skin thickness of the control area (*p* = 0.638). Furthermore, no difference in skin thickness was observed in patients with clinical features of cRISI in the irradiated and control areas (*p* = 0.345). The mean time after RT was 6.1 years. *Conclusions:* This study marks the first demonstration of epidermal barrier damage in patients in the long term following RT for head and neck cancer. The impairment of the epidermal barrier was observed independently of evident cRISI features. This observation underscores the necessity to recommend appropriate skin care, including the use of emollients, for all patients following RT. We also suggest that HF-USG examination is generally inconclusive in determining the degree of skin damage in the late period after RT.

## 1. Introduction

Radiotherapy (RT) constitutes an essential component of head and neck cancer therapy. Unfortunately, despite the intensive development of RT techniques in recent years, it still poses the risk of early and late side effects, manifesting as radiotherapy-induced skin injury (RISI, also known as radiation dermatitis or radiodermatitis), with the skin being the most commonly affected organ. 

Late reactions (chronic RISI; cRISI) may arise as early as 3 months post RT or even several years after treatment. These reactions stem from damage to epidermal and dermal cells induced by reactive oxygen species, resulting in an imbalance of pro-inflammatory and pro-fibrotic cytokines such as tumor necrosis factor (TNF), interleukin (IL)-6, IL-1, tumor growth factor beta (TGF-beta), platelet-derived growth factor (PDGF), and connective tissue growth factor (CTGF). Unlike early reactions, late reactions do not affect the effectiveness of therapy, but they can still significantly deteriorate patients’ quality of life. cRISI encompasses various morphologies (hyperpigmentation, vascular changes, alopecia, skin atrophy, dermal fibrosis, etc.) and is an irreversible and progressive condition [1,2,3]. In this context, the head and neck area seems to be particularly special because, in addition to RT-induced injury, it is constantly exposed to various exposomes such as ultraviolet radiation, cigarette smoke, air pollution, and weather conditions. 

In our previous studies, we have demonstrated skin biophysical abnormalities during RT in patients with head and neck cancer, regardless of clinically obvious acute RISI (aRISI) [4,5]. However, there are no studies in the literature regarding the impact of RT on the biophysical parameters of the skin (including the skin barrier) in the long term after completion of treatment.

### Aim

We hypothesized that RT contributes to long-term skin damage. Validating this hypothesis will enable the development of evidence-based principles for patient management post treatment. The aim of this study was to evaluate the biophysical properties of the skin in patients during the late period following RT for head and neck cancer (at least 90 days after the end of RT). 

## 2. Materials and Methods

This was a single-center non-randomized prospective study. The inclusion criteria consisted of (1) adult patients diagnosed with head and neck cancer, (2) patients treated with RT (with or without concomitant chemotherapy, and regardless of whether cRISI symptoms were present or absent), and (3) patients who completed RT at least 90 days prior to study recruitment. Exclusion criteria included (1) local treatment within the month preceding the study, (2) use of topical preparations (such as emollients or sunscreens) within 24 h before examination, and (3) patients undergoing RT for cancers other than head and neck tumors. Patients were recruited from the group of patients undergoing RT at the Greater Poland Cancer Center, offering participation in the study to every patient attending a follow-up visit who met the inclusion and exclusion criteria. Recruitment took place from May to August 2023. The control group comprised adult, healthy, non-smoking individuals. Ultimately, 16 patients and 15 healthy volunteers were included in the analysis. All measurements were conducted at the Department of Dermatology Poznan University of Medical Sciences.

Clinical assessment was based on the LENT-SOMA scale dedicated to late post-radiation reactions [6].

For evaluation of the biophysical properties of the skin, all patients underwent an analysis of transepidermal water loss (TEWL), stratum corneum hydration (SCH), and skin visualization using high-frequency ultrasonography (HF-USG). These measurements were performed and evaluated by the same non-blinded trained physician (dermatologist). TEWL was assessed with the use of Tewameter TM 300 (Courage-Khazaka, Cologne, Germany) according to the guidelines of the European Society of Contact Dermatitis [7]. At least 10 measurements, given as a mean value (expressed in SI units g/m^2^/h), were carried out. The normal range is estimated at 0–25 g/m^2^/h. Corneometry, evaluating SCH, was performed with the use of a corneometer CM 825 (Courage-Khazaka, Cologne, Germany). In accordance with the guidelines, six measurements were determined, given as a mean value in arbitrary units (a.u. range: 0–130). Skin thickness and the presence of subepidermal low echogenic band (SLEB) were assessed using HF-USG (Dermascan C ver. 3; Cortex Technology, Hadsund, Denmark), which was conducted with a 20 MHz probe with a resolution of 60 × 200 µm and a penetration depth of 15 mm. The images were saved as a B-mode presentation. Three measurements of skin thickness (epidermis and dermis) were taken within each sonogram, and the results were averaged.

Participants in both the study and control groups were instructed not to use any topically applied products (e.g., emollients, sunscreen, or make-up) for 24 h before evaluation. In the study group, assessments were conducted in the irradiated area of the neck and for control purposes: (1) in patients irradiated on only one side of the neck, in the contralateral location (7 patients), and (2) in patients where irradiation covered the entire surface of the neck, in the supraclavicular region (9 patients).

This study was conducted in accordance with the Declaration of Helsinki and was approved by the Poznan University of Medical Sciences Ethical Committee (No. 1013/12) and all patients provided written informed consent. 

### Statistical Analysis

The normality of distributions was assessed using the Shapiro–Wilk test. As many of the groups did not exhibit normal distribution, Mann–Whitney (MW) tests were performed. All statistical analyses were performed with the PAST v.2.09 software. Graphs were rendered using Plotly Chart Studio online.

## 3. Results

The characteristics of the study group, as well as clinical assessments based on the LENT-SOMA scale, are presented in Table 1. The average age of the control group was 59.6 years (range: 40 to 73), and the group comprised 5 women and 10 men. The study and control groups were matched for gender and age (*p* = 0.51).

**Figure 1:**
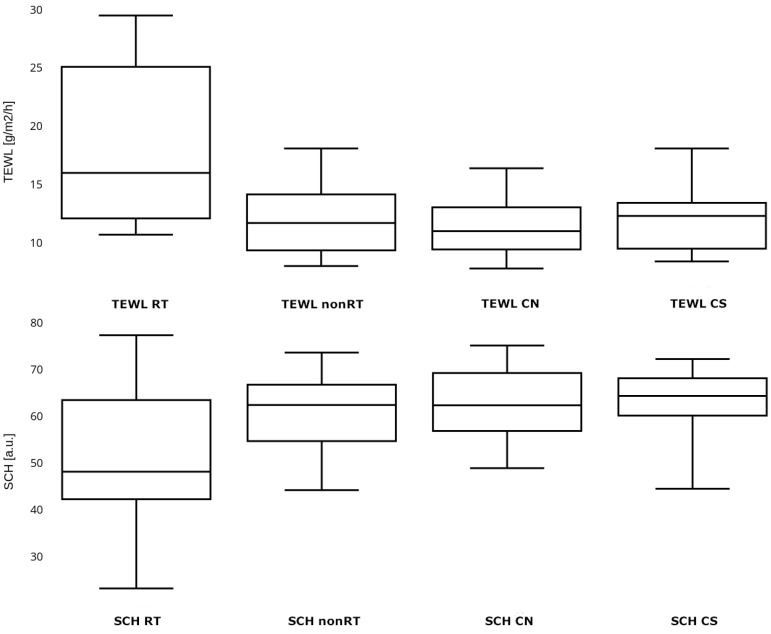
Transepidermal water loss and stratum corneum hydration in the study and control group. TEWL—transepidermal water loss, SCH—stratum corneum hydration, TEWL RT and SCH RT—parameters measured in the study group in the irradiated location, TEWL nonRT and SCH nonRT—parameters measured in the study group in the non-irradiated location, TEWL CN and SCH CN—parameters measured in the control group in the neck skin area, TEWL CS and SCH CS—parameters measured in the control group in the supraclavicular skin area.

The obtained results for TEWL and SCH are presented in Table 2 and Table 3, and those for skin thickness in Figure 1.

To verify whether the choice of the supraclavicular area of the skin, as a control site in patients undergoing bilateral irradiation of the neck, is appropriate and does not lead to the misinterpretation of the results, measurements were taken in both locations (neck skin and supraclavicular area) in individuals from the control group. No significant difference in TEWL and SCH measurements was found for the corresponding locations in healthy individuals (MW *p* = 0.534 and *p* = 0.034, respectively). There was also no significant difference between the TEWL of non-irradiated skin in individuals from the study group in the contralateral and supraclavicular locations (MW *p* = 0.958), nor between the hydration of the SCH in non-irradiated skin of individuals from the study group in the contralateral and supraclavicular locations (MW *p* = 0.318).

A significantly higher TEWL was observed in the irradiated area compared to the control area in the study group (MW *p* = 0.004). The SCH in the irradiated area was lower than in the non-irradiated area, but no statistically significant difference was observed (MW *p* = 0.073). There was no significant difference observed in the values of TEWL and SCH in the irradiated area between the group of patients with and without clinically obvious RISI (MW *p* = 0.192 and *p* = 0.415, respectively). The TEWL in the irradiated skin of patients without clinically obvious signs of RISI was significantly higher than in non-irradiated skin (MW *p* = 0.006), with no significant difference observed in the SCH (MW *p* = 0.438). A significantly higher TEWL was observed in the irradiated area compared to the TEWL in the neck and supraclavicular region in the control group (MW *p* = 0.001 and *p* < 0.001, respectively). There was no significant difference observed in the SCH in the irradiated area compared to the SCH in the neck and supraclavicular region in the control group (MW *p* = 0.021 and *p* = 0.031, respectively). The concomitant chemotherapy did not affect the values of TEWL and SCH, either in the skin subjected to radiation therapy or in the non-irradiated skin. The obtained *p*-values were as follows: TEWL in the irradiated area for patients who received chemotherapy vs. patients without chemotherapy (MW *p* = 0.525), TEWL in the non-irradiated area for patients who received chemotherapy vs. patients without chemotherapy (MW *p* = 0.397), SCH in the irradiated area for patients who received chemotherapy vs. patients without chemotherapy (MW *p* = 0.915), and SCH in the non-irradiated area for patients who received chemotherapy vs. patients without chemotherapy (MW *p* = 0.112). Since all patients in the study group, except one, exhibited features of aRISI during RT, statistical analysis regarding the potential impact of aRISI on the state of the epidermal barrier in the distant period was discontinued.

**Figure 2:**
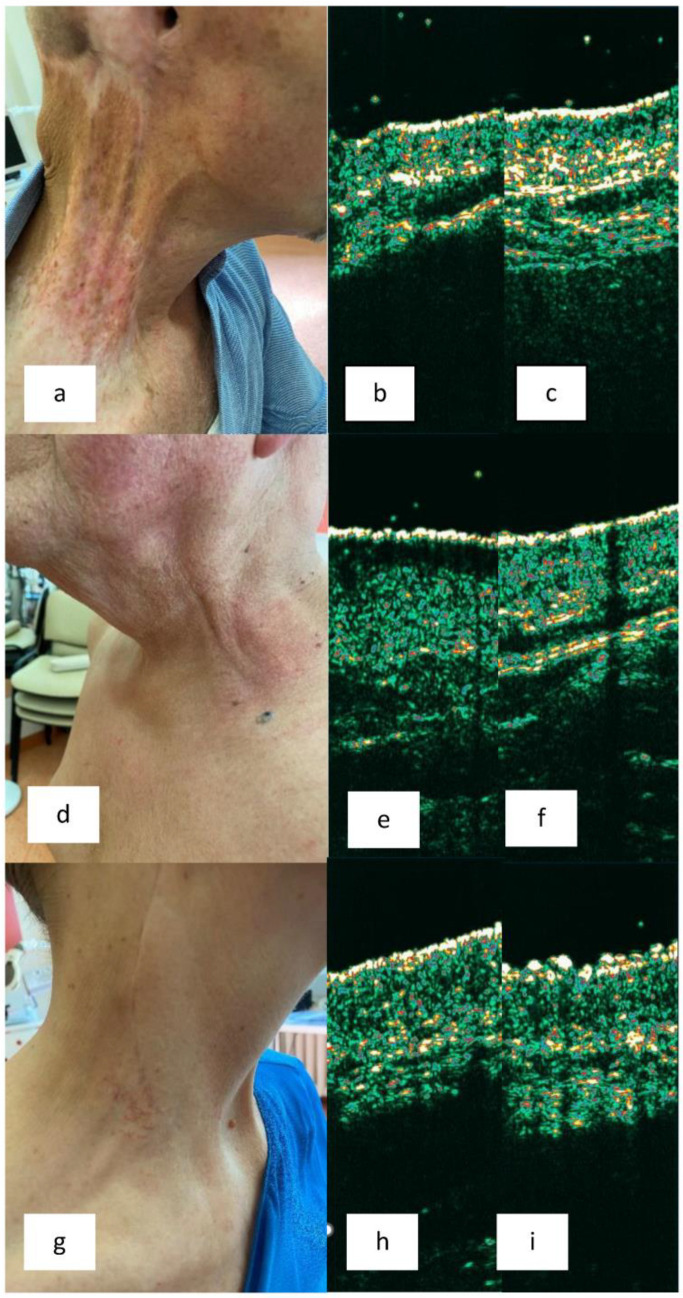
Clinical presentations and their corresponding skin sonograms of the irradiated and control areas. (**a**) Patient with BCC of the external ear with lymph node metastases. 2 years post unilateral radiotherapy (RT). Grade 3 of chronic radiation induced skin injury (cRISI): atrophy, erythema, discoloration, limited mobility. (**b**,**c**) Sonogram of irradiated skin (**b**) and contralateral non-irradiated skin (**c**). Skin thinning is visible. (**d**) Patient with tonsil cancer with lymph node metastases. 5 years post bilateral RT. Grade 2 cRISI: erythema and swelling. (**e**,**f**) Sonogram of irradiated skin (**e**) and surrounding non-irradiated skin (**f**). Subepidermal low echogenic band (SLEB) is visible. (**g**) Patient with melanoma in the temporoparietal region with lymph node metastases. 6 years post unilateral RT. Grade 1 cRISI: slight atrophy, telangiectasia, subjective symptoms. (**h**,**i**) Sonogram of irradiated skin (**h**) and contralateral non-irradiated skin (**i**). Flattened entry echo and slight skin thinning are visible.

Taking into account the entire group, the skin thickness of the irradiated area did not differ significantly from the skin thickness of the control area (MW *p* = 0.638). The skin in the irradiated area in patients with clinically evident signs of cRISI was thinner than the skin in the control area; however, this difference was not statistically significant (MW *p* = 0.345). The same situation was observed when considering only a group of patients with clinically obvious skin atrophy (MW *p* = 0.21). There were also no statistically significant differences in skin thickness observed when analyzing subgroups of patients with erythema and skin hardening (MW *p* = 0.885 and *p* = 0.697 respectively). SLEB was observed in only one patient. This was a patient with features of cRISI characterized by erythema. Figure 2 presents sample clinical photographs and their corresponding skin sonograms of the irradiated and control areas.

## 4. Discussion

A compromised epidermal barrier is widely considered one of the components of RISI. However, studies demonstrating this condition have primarily focused on its acute form, predominantly in the breast area [5,8,9,10]. It is noteworthy that, compared to breast skin, the skin of the head and neck area is subject to a broader array of factors that can further compromise the skin barrier, including ultraviolet radiation, cigarette smoke, air pollution, and weather conditions. Despite the lack of evidence from studies, there is a general consensus that proper skincare with emollients is the mainstay of care for patients with cRISI [2,11]. To the best of our knowledge, this study represents the first attempt to objectively assess skin condition years after undergoing RT (the mean time after RT was 6.1 years). For this purpose, we selected two biophysical parameters, TEWL and SCH, which reflect various aspects of skin condition. TEWL is considered an indicator of the state of the epidermal barrier, while SCH is used to assess the degree of skin dryness [12]. We observed significantly higher TEWL values in the area subjected to RT compared to the skin surrounding the irradiated area (*p* = 0.004) and in comparison to the TEWL of the control group. Furthermore, the TEWL value was higher in the irradiated area, even in those patients where clinically evident signs of RISI were not observed. The SCH values were lower in the irradiated area compared to the results obtained from the surrounding skin, but the difference did not reach statistical significance (*p* = 0.073). Additionally, we excluded the influence of concomitant chemotherapy on TEWL and SCH in the study group. Thus, for the first time in the literature, we confirmed the persistent damage to the epidermal barrier in patients undergoing RT in the late period after the completion of treatment, regardless of clinically evident signs of cRISI. A skin barrier defect is a common element in the pathogenesis of several dermatoses, including atopic dermatitis and other forms of eczema. In these conditions, proper skin care is recommended as first-line therapy to restore the skin’s barrier function and reduce the likelihood of further damage. Additionally, the role of emollients as steroid-sparing agents is emphasized [13,14]. Therefore, explaining to the patient the principles of proper skin care seems to be an integral part of managing a patient after RT.

In the literature, there are several publications dedicated to the ultrasonographic assessment of skin subjected to RT years after the completion of therapy [15,16,17,18,19,20,21]. Most of them focus on the breast area of the skin. Results differ significantly among the authors. In the first publication, Warszawski et al. [15] demonstrated an increase in skin thickness in the late period after RT in patients with breast cancer. Similarly, studies evaluating skin with features of edema and/or induration showed thickening compared to healthy skin [20,21]. On the other hand, Wong et al. [19] observed a reduced skin thickness in the irradiated breast compared to the contralateral non-irradiated area. The skin of the contralateral area appears to be the most reliable control site, as skin thickness depends on various factors such as gender, age, anatomical location, ethnicity, and even the time of day [22,23]. For this reason, interpersonal comparisons may be less reliable. The contralateral skin is usually easily accessible in the case of breast cancer. Unfortunately, in the case of head and neck tumors, bilateral lymph node irradiation is often necessary. Therefore, in our studies, in such situations (nine patients), we performed the control measurement in the supraclavicular area. In contrast to previous publications, we did not observe differences in skin thickness regardless of the presence or absence of cRISI symptoms. Analyses within subgroups characterized by specific cRISI symptoms (in order: atrophy, erythema, and skin hardening) also did not reveal statistically significant differences in skin thickness, although a lesser skin thickness was observed in all subgroups with clinically evident lesions in irradiated compared to non-irradiated areas, in contrast to subgroups without clinically evident cRISI features. Of course, it should be emphasized that the examined subgroups were sometimes very small (e.g., only two individuals with clinically evident skin hardening), which constitutes a notable limitation of our study. On the other hand, it is also worth noting that in the assessment of individual patients, we observed differences in the sonograms of the skin subjected to RT and the control area (Figure 2). In one case characterized by erythema, SLEB (which serves, among other things, as a marker of inflammatory infiltration within the skin [23]) was visualized. In our opinion, significant discrepancies in the obtained sonograms of patients in the distant period after RT reflect the diverse clinical presentations of cRISI, as confirmed by the LENT-SOMA scale, and therefore, these should be interpreted individually in relation to the patient’s clinical condition and compared to the surrounding or contralateral skin.

The main limitation of the study is the small study group and small subgroups, which complicates the conduct of certain analyses, such as multivariate analysis. However, we hope that the presented results will initiate larger, preferably multicenter studies. Additionally, a certain difficulty was the lack of accessibility to the non-irradiated contralateral area for all patients. As mentioned above, in such situations, we assessed the skin of the supraclavicular area.

## 5. Conclusions

In summary, in our studies, for the first time, we demonstrated persistent epidermal barrier damage in patients who underwent RT years earlier. Impairment of the epidermal barrier was observed independently of evident cRISI features. These results confirm the necessity of implementing proper skin care, including syndets, emollients, and photoprotection, as the primary therapy for all patients after RT, including those without clinical signs of skin damage. We also suggest that one cannot usually draw conclusions about the degree of cRISI based on HF-USG examination. However, the assessment of HF-USG in patients in the long term after RT can be helpful, but it should be performed in correlation with the individual clinical presentation.

## Figures and Tables

**Table 1:** The characteristics of the study group.

Feature	No of Patients (Total 16)
female/male	5/11
mean age (min–max) [years]	62.5 (37–80)
type of neoplasm	1 SCC of the tongue base
	2 SCC of the tongue base and floor of the mouth
	2 SCC of larynx
	4 SCC of tonsil
	2 adenoid cystic carcinoma of the parotid gland
	2 adenocarcinoma of the parotid gland
	3 lymph node metastasis (melanoma, BCC)
RT	RT technique: IMRT (all patients)
	Dose pre-fraction: 2 Gy
	Dose for tumor bed: 62–70 Gy
	Dose for high-risk lymph nodes: 60 Gy
	Dose for elective lymph nodes—50 Gy
aRISI	grade 0—1 patients
	grade I—3 patients
	grade II—9 patients
	grade III—3 patients
	grade IV—0 patients
concomitant chemotherapy	9
mean time post RT (min–max) [years]	6.1 (1–13)
cRISI grade	grade 0—6 patients
	grade I—5 patients
	grade II—2 patients
	grade III—3 patients
	grade IV—0 patients

aRISI—acute radiotherapy-induced skin injury, BCC—basal cell carcinoma, cRISI—chronic radiotherapy-induced skin injury, RT—radiotherapy, SCC—squamous cell carcinoma.

**Table 2:** TEWL and SCH in study and control groups and subgroups.

Parameter	Study Group						Control Group	
	Irradiated Area			Control Area			Neck	Supra-Clavicular
	Total	Grade 0 Subgroup *	Grade I–III Subgroup *	Total	Contra-Lateral	Supra-Clavicular		
	n = 16	n = 6	n = 10	n = 16	n = 8	n = 8	n = 15	n = 15
TEWL [g/m^2^/h] Median (min–max) [95% CI]	16.0 (10.7–29.5) [14.9–22.0]	21.6 (11.8–29.5)	14.2 (10.7–27.5)	11.7 (8.0–18.1) [10.4–13.6]	11.7 (8.0–18.1)	11.2 (8.6–16.2)	11.0 (7.8–16.4) [10.0–12.7]	12.3 (8.4–18.1) [10.6–13.5]
SCH [a.u.] Median (min–max) [95% CI]	48.2 (23.2–77.4) [44.1–60.3]	54.1 (40.1–77.4)	47.7 (23.2–75.2)	62.5 (44.2–73.7) [56.1–65.4]	58.1 (44.2–73.7)	63.4 (55.1–72.5)	62.4 (48.9–75.2) [58.5–67.4]	64.4 (44.5–72.3) [58.4–67.1]

* grade of chronic radiotherapy-induced skin injury according to the LATE-SOMA scale; TEWL—transepidermal water loss, SCH—stratum corneum hydration, a.u.—arbitrary units.

**Table 3:** Skin thickness in study and control groups and subgroups.

	Total	cRISI Grade *		Clinically Evident Atrophy		Clinically Evident Erythema		Clinically Evident Skin Hardening	
		Grade 0	Grade I–III	No	Yes	No	Yes	No	Yes
	n = 16	n = 6	n = 10	11	5	12	4	14	2
Skin thickness [mm]	Irradiated area								
	1.912 (1.361–3.815) 95% CI [1.66 to 2.36]	1.639 (1.401–2.021)	2.031 (1.361–3.815)	1.669 (1.361–3.815)	2.134 (1.907–2.702)	1.689 (1.361–2.832)	2.418 (1.907–3.815)	1.808 (1.361–3.815)	2.31 (1.917–2.702)
	Control area								
	1.98 (1.262–3.249) 95% CI [1.81 to 2.6]	1.584 (1.321–2.132)	2.844 (1.262–3.249)	1.608 (1.262–3.16)	3.001 (1.828–3.249)	1.649 (1.262–3.249)	2.863 (1.828–3.05)	1.759 (1.262–3.249)	2.857 (2.673–3.04)

* the grade of chronic radiotherapy-induced skin injury according to the LATE-SOMA scale.

## Data Availability

To obtain access to supported data, please contact the corresponding author.
